# Actin Dysregulation Mediates Nephrotoxicity of Cassiae Semen Aqueous Extracts

**DOI:** 10.3390/toxics12080556

**Published:** 2024-07-30

**Authors:** Jinlan Yang, Sheng Xiao, Ludi Li, An Zhu, Wusheng Xiao, Qi Wang

**Affiliations:** 1 Department of Toxicology, School of Public Health, Peking University, Beijing 100191, China; yangjinlan@bjmu.edu.cn (J.Y.); sheng_xiao@pku.edu.cn (S.X.); liludi@bjmu.edu.cn (L.L.); zhuan@fjmu.edu.cn (A.Z.); wxiao@bjmu.edu.cn (W.X.); 2 Key Laboratory of State Administration of Traditional Chinese Medicine (TCM) for Compatibility Toxicology, Peking University, Beijing 100191, China; 3 Beijing Key Laboratory of Toxicological Research and Risk Assessment for Food Safety, Peking University, Beijing 100191, China

**Keywords:** *Cassiae* semen, nephrotoxicity, RhoA–ROCK pathway, cytoskeleton

## Abstract

*Cassiae* semen, commonly consumed as roasted tea, has been widely used for both medicinal purposes and dietary supplements. In this study, we investigated the nephrotoxic effects and underlying mechanisms of *Cassiae* semen aqueous extracts (CSAEs) using computational and animal models. Both male and female Sprague Dawley rats were treated with 4.73–47.30 g/kg (body weight) of CSAEs by oral gavage twice a day for 7–28 days. We found that serum and urinary biomarkers of kidney injury and kidney coefficients were increased in a dose-dependent manner, and were accompanied by morphological alterations in the kidneys of CSAEs-treated rats. Computational and molecular docking approaches predicted that the three most abundant components of CSAEs—obtusifolin, aurantio-obtusin, and obtusin—exhibited strong affinity for the binding of F-actin, ROCK1, and Rac1, and the RhoA–ROCK pathway was identified as the most likely regulatory mechanism mediating the nephrotoxicity of CSAEs. Consistently, immunofluorescence staining revealed F-actin and cytoskeleton were frequently disturbed in renal cells and brush borders at high doses of CSAEs. Results from gene expression analyses confirmed that CSAEs suppressed the key proteins in the RhoA–ROCK signaling pathway and consequently the expression of F-actin and its stabilization genes. In summary, our findings suggest that Cassiae semen can depolymerize and destabilize actin cytoskeleton by inhibition of the RhoA–ROCK pathway and/or direct binding to F-actin, leading to nephrotoxicity. The consumption of *Cassiae* semen as a supplement and medicine warrants attention.

## 1. Introduction

*Cassiae* semen, a herb consumed as a roasted tea and a class of traditional Chinese medicine (TCM), is widely used in eastern Asia for weight control and treatment of hyperlipidemia, dizziness, constipation, and eye diseases [1,2,3,4]. Traditionally, *Cassiae* semen is defined as a low toxic agent [4]. However, the safety of *Cassiae* semen remains controversial. For example, hepatotoxicity has been reported in rats after a 90-day administration of extract mixed into the fodder [5]. Nephrotoxicity manifested by pigment deposition, atrophy, or regeneration of renal tubules has also been found in rats after administration of *Cassiae* semen (10 g/kg body weight (B.W.)) [6]. These lines of evidence support the necessity to reevaluate the safety of *Cassiae* semen.

To date, over 70 components have been isolated and identified from *Cassiae* semen, approximately 53 of which are biologically active and structurally distinct anthraquinones (AQs) [1]. We previously revealed that after oral administration of *Cassiae* semen, obtusifolin, *Epi*-9-dehydroxyeurotinone-*β*-D-glucopyranoside (EDG), aurantio-obtusin, obtusin, 2-O-methyl-9-dehydroxyeurotinone (OMD), rhein, questin, emodin, and cassiaside were the nine most abundant components in plasma of rats [7]. Aurantio-obtusin, one major AQ ingredient of *Cassiae* semen, was shown to induce hepatotoxicity through enhanced reactive oxygen species (ROS) production, apoptosis, and ferroptosis [8]. Qin et al. found that after administration of aurantio-obtusin, a major anthraquinone compound of *Cassiae* semen at a dose of 200 mg/kg B.W., metabolites of aurantio-obtusin were detected and identified in the heart, liver, spleen, lungs and kidneys [9]. In a tissue distribution study of obtusifolin, after intragastric administration of 1.3 mg/kg B.W., obtusifolin was rapidly distributed into tissues, with the highest distribution in the liver, followed by lung, heart, kidney, spleen, and brain [10]. Yang et al. also observed the presence of chrysophanol, emodin, aloe-emodin, rhein, physcion, obtusifolin, and aurantio-obtusin in plasma after oral administration 1.25 g/kg B.W. of *Cassiae* semen extract, and the time to reach the maximum plasma concentration varied from 0.167 to 0.5 h [11]. Nephrotoxicity can be another problem of *Cassiae* semen, and the toxic molecules are worth exploring. Some components of TCMs, such as aristolochic acids, alkaloids, anthraquinones, and flavonoids, have been reported to cause kidney injury [12,13]. An adverse outcome pathway (AOP) identified loss of tubular cells and tubular vacuolization as key events in kidney toxicity [14]. Other mechanisms were also involved, such as the direct perturbations of cellular or subcellular organelle function, cell death (apoptosis and necrosis), calcium dysregulation and cytoskeleton malfunction [6]. Since the form of aqueous extracts mimics more closely its consumption as roasted tea and decoction, it is worthwhile to study the influences of *Cassiae* semen aqueous extracts (CSAEs) on kidney functions and the potential mechanisms.

Actin is one of the most abundant cytoskeleton proteins to support cellular structure and functions [15]. Actin cytoskeletal dynamics, which regulates intracellular calcium (Ca^2+^) concentration, is controlled by nuclear factor of activated T cells (NFAT)-dependent transcriptional activity [16] and by Rho GTPases via the RhoA–ROCK signaling pathway [17,18]. More importantly, cytoskeletal dynamics is also involved in cell death pathways, such as apoptosis [19] and necrosis [20]. It has been reported that actin destabilization is one of the factors mediating nephrotoxicity [21]. However, the intrinsic interaction between the main ingredients of CSAEs and cytoskeleton and their role in the renal toxicity of *Cassiae* semen remains obscure.

In this study, we aimed to elucidate the dose- and time-dependent effects and their underlying mechanisms of CSAEs-induced nephrotoxicity in rat models and computational approaches.

## 2. Materials and Methods

### 2.1. Chemicals and Materials

Ultrapure water was prepared by a Milli-Q system (Millipore, Bedford, MA, USA). Creatinine (Cr) and blood urea nitrogen (BUN) assay kits were purchased from Sichuan Maccura Biotechnology Co., Ltd. (Chengdu, China). Anti-ROCK1 (phospho T455 + S456; Cat. # ab97592), anti-myosin light chain (phospho S20; Cat. # Ab2480), anti-cofilin (phospho S3; Cat. # ab 12866), and F-actin staining kit (green fluorescence; Cat. # ab112125) were purchased from Abcam (Cambridge, UK). Anti-RhoA (26C4) (Cat. # sc-418) and anti-GAPDH (Cat. # A19056) were purchased from Santa Cruz Biotechnology (Santa Cruz, CA, USA) and Abclonal (Wuhan, China), respectively.

### 2.2. Preparation of Cassiae Semen Aqueous Extracts (CSAEs)

*Cassiae* semen (Anguo Qi An Pharmaceutical Co., Ltd., Anguo, China) was prepared using the seeds of *Senna obtusifolia* (L.) H. S. Irwin & Barneby (*S. obtusifolia*). Specifically, the seeds of *S. obtusifolia* were immersed in eightfold volumes of distilled water for 30 minutes (min) and then boiled for 60 min. The residue was mixed with fourfold volumes of distilled water, decocted for 30 min, and the entire process was repeated twice. All the collected supernatants were filtered through eight layers of gauze and condensed to obtain different concentrations of CSAEs (0.158, 0.525, and 1.577 g/mL).

### 2.3. Animal Treatment

A total of 32 male and 32 female Sprague Dawley (SD) rats (180–200 g) aged 8–12 weeks were obtained from the Department of Laboratory Animal Science at Peking University Health Science Center (Beijing, China). These rats were randomly divided into four treatment groups, with eight rats/sex/group. Typically, the dose level for a toxicology study should be 10- to 30-folds that of the therapeutic dose to ensure the safety of human consumption. The doses we used are based on the following calculations. According to the guidelines in the *Chinese Pharmacopoeia* (2020), the intake of *Cassiae* semen is limited to 15 g per day: 15 g/60 kg = 0.25 g/kg for humans (the average body weight of an adult is assumed as 60 kg). For the adjustment of interspecies difference (human-to-animal) in body size, a parameter value of 6.3 was used (body weight approach). Therefore, the equivalent dose for rats is 0.25 × 6.3 = 1.575 g/kg. To mimic the long-term and low-dose exposure in humans using a short-term animal toxicity study, we chose the 3-, 10- and 30-fold equivalent doses, which were equivalent to 4.73 g/kg, 15.75 g/kg, and 47.30 g/kg. All animals were administered by gavage twice a day for 28 consecutive days. Control animals were treated with solvent (distilled water). Animals were housed under a controlled light intensity of 200 Lx with a 12 h/12 h light/dark cycle, temperature of 25 °C ± 1 °C, and 55–60% humidity. All experimental procedures were conducted according to an approved protocol by the Animal Experimental Ethical Committee of Peking University (LA2017227, Beijing, China) and under the guidelines of the Association for Assessment and Accreditation of Laboratory Animal Care International (AAALAC).

### 2.4. Serum Biochemical Parameters

Elevated levels of Cr and BUN are the most commonly used markers of kidney injury. Serum Cr and BUN levels were detected on days 0, 7, 14, and 28 of CSAEs treatment using a serum biochemistry analyzer (Hitachi, Tokyo, Japan). Blood was collected via the orbital venous plexus, kept at room temperature for 30 min for coagulation, and then centrifuged at 3000× *g* for 10 min (1630 RCF, MF300, Incheon, Republic of Korea).

### 2.5. Urinalysis

The urinary calbindin, kidney injury molecule 1 (KIM-1), osteopontin (OPN), neutrophil gelatinase-associated lipocalin (NGAL), cystatin C, and β-2-microglobulin (β2M) levels were early indications of kidney injury. Urinary samples were collected 12 h after the last treatment on the day, immediately centrifuged at 3000× *g* for 10 min (1630 RCF, MF300, Incheon, Republic of Korea), and then tested by a Milliplex Luminex 200TM and Milliplex^®^ Map Kit following the manufacturer’s instructions (Cat. # RKTX2MAG-37K and RKTX1MAG-37K, EMD Millipore, Billerica, MA, USA). Calbindin, KIM-1, and OPN levels were tested on days 7, 14, and 28, and β2M, cystatin C, and NGAL/lipocalin 2 levels were tested on day 28 of CSAEs treatment.

### 2.6. Histopathological Examinations

Rats were sacrificed on day 28 and the left kidney were collected. Kidneys were fixed in 4% paraformaldehyde, sequentially dehydrated with ethanol and xylene, embedded in paraffin, sectioned at 5 μm thickness, and stained with hematoxylin and eosin (H&E) for histology. The H&E-stained kidney sections were analyzed under an optical microscope (Olympus, Tokyo, Japan) to examine morphological changes.

### 2.7. Fluorescent Staining of Cellular Cytoskeleton

Kidneys embedded in paraffin were sliced into sections of 4 μm thickness, deparaffinized and hydrated, and then rinsed in distilled water and phosphate-buffered saline (PBS) 3 times. Tissue sections were incubated in 0.5% Triton X-100 for 30 min to allow permeabilization and then with green fluorescent phalloidin conjugate for 1 h followed by rinsing with 1× PBS. Images were recorded using a confocal laser-scanning microscope (Nikon, Tokyo, Japan) with an FITC emission filter (488/520 nm).

### 2.8. Molecular Docking

Cytoscape (version 3.7.2) software was used to construct a composition–target interaction network model with the nine most abundant components of CSAEs identified in rat blood and key proteins of four renal injury pathways. Nodes in the network represent compounds or proteins. If a protein is a potential target for a specific CSAEs compound, the nodes are linked by edges.

**Figure 1:**
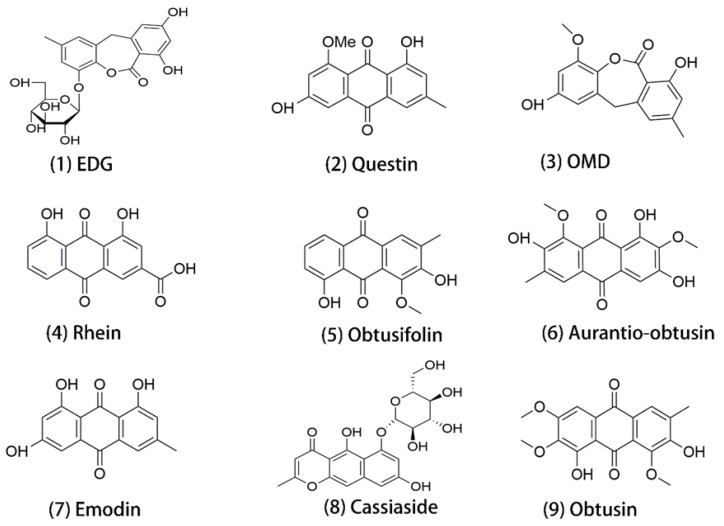
Structures of the 9 major components identified in rat plasma after 28 days of CSAEs administration. EDG, *Epi*-9-dehydroxyeurotinone-*β*-D-glucopyranoside; OMD, 2-*O*-methyl-9-dehydroxyeurotinone.

To explore the potential mechanism of kidney injury caused by CSAEs, the key proteins of different kidney injury pathways (apoptosis, necrosis, calcium regulation, and cytoskeleton regulation) were the central focus, and the 3D structures of these proteins were retrieved from the Protein Data Bank (PDB). The interactions between these proteins and the nine major components in CSAEs (Figure 1) were predicted using SYBYL-X 2.0 software (Tripos, St. Louis, MO, USA). Before docking, the proteins were prepared by removal of water molecules, metal ions, and solvent molecules, repair of side chains and side chain amides, and addition of hydrogen atoms. The nine major components, the ligands of proteins, were prepared by the addition of Gasteiger–Hückel charges in the Tripos force field to attain minimum energy status. Using SYBYL-X 2.0 software, a total score was used as a comprehensive evaluation of hydrophobic complementarity, polar complementarity, solvation terms, and entropic terms. When a total score is higher than 7, the interaction between the protein and molecule is considered as stable.

### 2.9. RNA Isolation and RT-qPCR

Quantitative reverse-transcription polymerase chain reaction (RT-qPCR) was performed to examine the expression of genes related to the RhoA–ROCK pathway. Total ribonucleic acid (RNA) was isolated from kidney tissues using TRIzol reagent (Invitrogen, Carlsbad, CA, USA). Subsequently, complementary deoxyribonucleic acid (cDNA) was synthesized from 10 μL of total RNA using a reverse-transcriptase reaction. To quantify the relative messenger RNA (mRNA) expression, the primers were synthesized, as shown in Table S1, with *Gapdh* used as the internal reference. Relative mRNA expression was calculated using the 2^−ΔΔCt^ method, and the fold change was normalized to the expression levels of control animals.

### 2.10. Western Blotting

Total protein was extracted from kidneys by RIPA buffer supplemented with proteinase inhibitors and phosphatase inhibitors and then centrifuged at 14,000× *g* for 5 min at 4 °C. The protein concentration in the supernatant was measured using the BCA method and denatured at 100 °C for 10 min. Equal amounts of protein (15 μg) were separated on a 4–15% precast gel and then electrophoretically transferred to polyvinylidene difluoride membranes at 220 mA for 120 min. The membranes were blocked with 5% bovine serum albumin in Tris-buffered saline with 0.1% Tween 20 (TBST) buffer for 120 min at room temperature and then rinsed three times in TBST for 10 min. The membranes were incubated with primary antibodies in TBST overnight at 4 °C, and then blots were probed by incubation with a secondary antibody for 120 min at room temperature. Finally, the proteins were visualized using ECL chemiluminescence solution and imaged by a Tanon 4500 System (Tanon, Shanghai, China).

### 2.11. Statistical Analysis

Data analyses were performed using SPSS 22.0 software (IBM, New York, NY, USA). All data are presented as means ± standard deviation (SD), and statistical significance was tested by one-way analysis of variance (ANOVA) followed by a least-significant difference test (LSD) for multiple comparisons as appropriate. A *p* value < 0.05 was considered statistically significant.

## 3. Results

### 3.1. Administration of CSAEs Induces Nephrotoxicity in Rats

**Figure 2:**
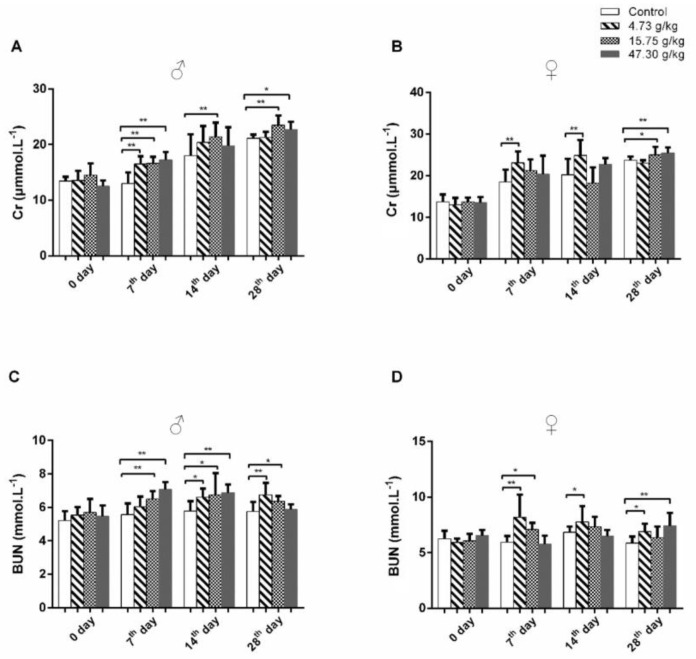
CSAEs increased serum Cr and BUN levels in rats. Male and female SD rats were treated with CSAEs (4.73, 15.75, and 47.3 g/kg/day) for 7–−28 days. Serum was collected after the final treatment at each time point. (**A**,**B**) Serum Cr levels in male (**A**) and female (**B**) rats; (**C**,**D**) serum BUN levels in male (**C**) and female (**D**) rats. * and ** indicate *p* < 0.05 and *p* < 0.01, respectively, when compared with the vehicle control rats; *n* = 8 in each sex.

To evaluate the effects of *Cassiae* semen on renal functions, rats were treated with different doses of CSAEs for 7–28 days. Renal injury was examined by the measurement of serum and urine biomarkers. Increases in Cr and BUN are critical manifestations of acute kidney injury. We did not observe obvious dose- or time-dependent effects in either male or female rats. However, when compared to the control group at days 7, 14, and 28, the levels of Cr and BUN in male rats treated with medium dose and the BUN levels in female rats treated with low dose were significantly elevated (Figure 2). According to the RIFLE criteria, a 1.5-fold increase in Cr level indicates an increased risk of renal injury and a 2-fold increase indicates renal injury [22]. We thus postulated that CSAEs may cause mild damage to kidneys, which led us to further investigate using urinalysis.

**Figure 3:**
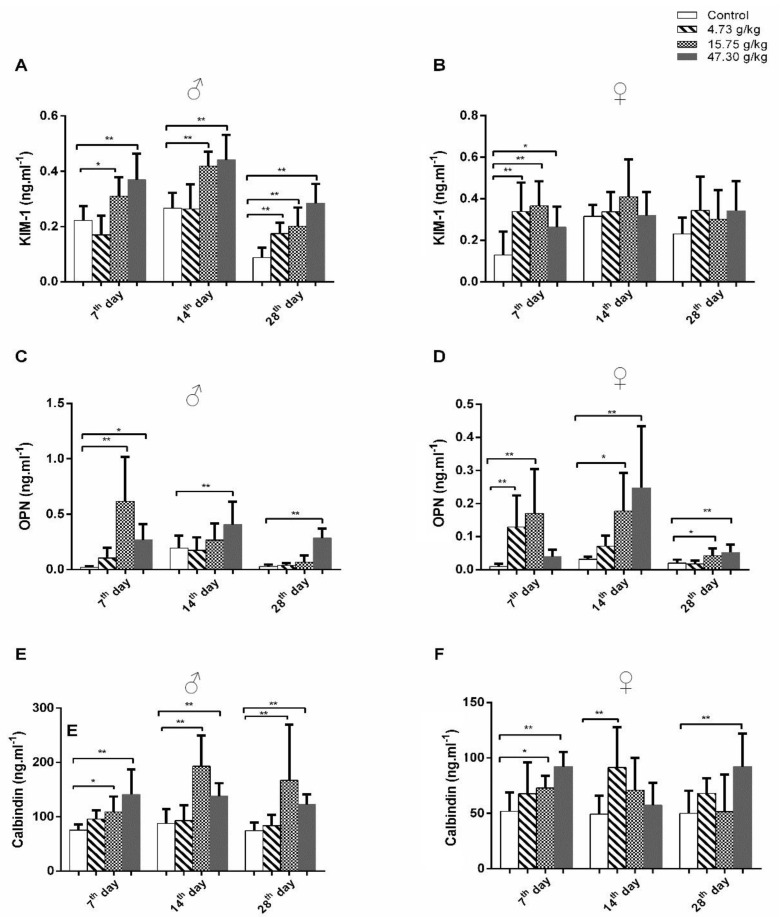
CSAEs increased urinary KIM−1, OPN, and calbindin protein levels in rats. Male and female SD rats were treated with CSAEs (4.73, 15.75, and 47.3 g/kg/day). Urine was collected after the final treatment on days 7, 14, and 28. (**A**,**C**,**E**) Levels of KIM−1, OPN, and calbindin in male rats; (**B**,**D**,**F**) results of the female rats. * and ** indicate *p* < 0.05 and *p* < 0.01, respectively, when compared with the vehicle control rats; *n* = 8 in each sex.

**Figure 4:**
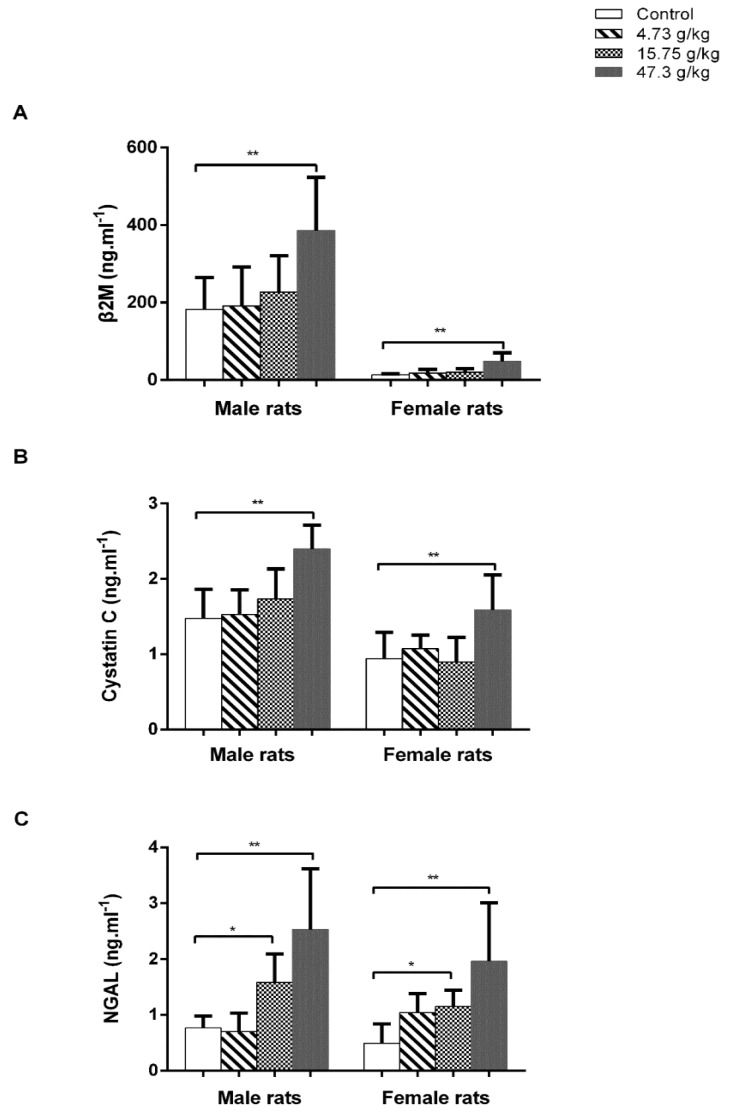
CSAEs−induced elevation in β2M, cystatin C, and NGAL levels in urine. Male and female SD rats were treated with AECS (4.73, 15.75, and 47.3 g/kg/day). Urine was collected after the final treatment on day 28. β2M (**A**), cystatin C (**B**), and NGAL (**C**) levels were measured by assay kits. * and ** indicate *p* < 0.05 and *p* < 0.01, respectively, when compared with the vehicle control rats; *n* = 8 in each sex.

Then, we analyzed urinary levels of renal damage marker proteins KIM-1, OPN, calbindin, β2M, cystatin C, and NGAL (Figure 3 and Figure 4, Table S2). Compared to control rats, urinary levels of KIM-1, OPN, and calbindin proteins were markedly increased in male rats treated with 15.75 or 47.30 g/kg of CSAEs for 7 to 28 days (Figure 3A,C,E). In female rats, a significant rise in KIM-1 protein level was noted at day 7 of CSAEs treatment, which became comparable with control animals at later time points (Figure 3B). By contrast, similar to male rats, the urinary levels of OPN and calbindin proteins remained significantly higher for all three treatment durations when compared to time-matched control rats (Figure 3D,F).

We further determined the urinary levels of β2M, cystatin C, and NGAL on day 28 of CSAEs treatment. Results showed that treatment of CSAEs, particularly at the highest dose (47.30 g/kg), caused obvious increases in the levels of these three marker proteins in both male and female animals (Figure 4). Interestingly, we noted a significant difference in the urinary β2M levels between male and female rats at basal and CSAEs-treated conditions, while there was no such difference for cystatin C and NGAL proteins (Figure 4). Tsuji et al. compared the urinary biomarker excretion levels between male and female rats at 5, 7, 9, and 12 weeks of age, and revealed higher excretion levels of β2-microglobulin (β2-MG) in male rats than female rats [23], in line with our findings. Despite such sex differences in absolute amounts, both male and female rats showed the same pattern, but with changes by different extents in nephrotoxicity markers after the administration of CSAEs. Together, these data demonstrate that high doses of CSAEs treatment cause renal damage as early as day 7 in both male and female rats.

### 3.2. Changes in Organ Coefficients and Histopathology by CSAEs Administration

**Figure 5:**
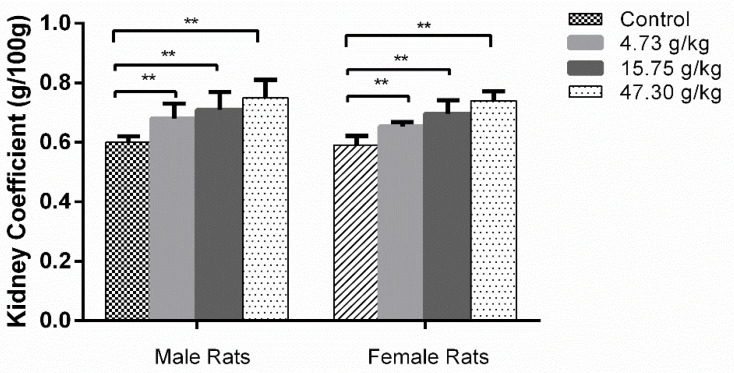
Kidney coefficients of male and female rats after a 28-day repeated oral dose toxicity study of AECS. ** *p* < 0.01 compared with the control (*n* = 8).

As presented in Figure 5, compared to the control group, the organ coefficients of the kidneys were significantly increased in all treatment groups of both male and female rats. These increased approximately 25% in male and female rats treated with the highest dose (47.30 g/kg) of CSAEs.

**Figure 6:**
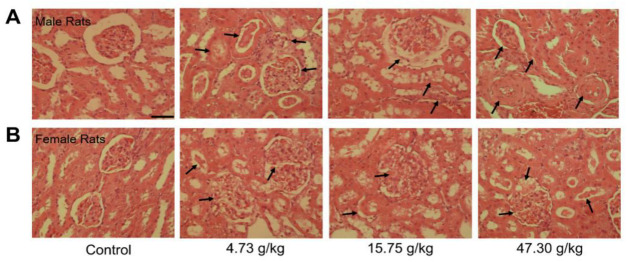
Representative histopathological images of male (**A**) and female (**B**) rats after administration of CSAEs for 28 days. Arrows indicate the damaged glomerulus tubes. Scale bar = 50 μm; *n* = 8.

The histopathological changes in kidney tissues after CSAEs treatment were presented in Figure 6. Compared to the well-characterized normal kidney tissue structures in control rats, morphological changes in the kidney were evident in all treatment groups and both sexes, as evidenced by larger and swelling glomeruli, shrunken or even disappearance of glomerular capsules, tubular dilatation and congestion, and the presence of protein cast or granular cast in the renal tubular lumen. Thus, these results also support the presence of renal toxicity in CSAEs-treated rats. As there is no obvious difference between male and female rats in most biochemical and histopathological markers, only male rats were employed for the following mechanistic investigation.

### 3.3. Molecular Docking Predicts the Potential Mechanisms of CSAEs-Induced Kidney Injury

**Figure 7:**
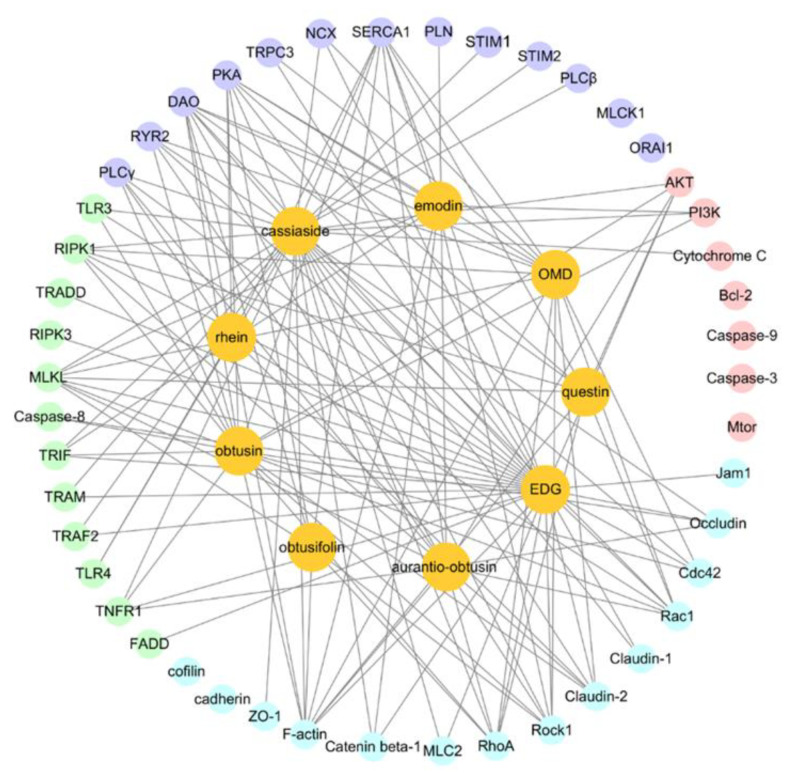
Cytoscape shows the interactions between 9 major components of CSAEs and 4 known kidney injury pathways. The RhoA–ROCK pathway, apoptosis pathway, calcium regulation pathway, and necrosis pathway are represented by blue, pink, purple, and green, respectively.

To examine the underlying mechanisms mediating CSAEs-induced nephrotoxicity, we applied an in silico approach to evaluate the molecular interactions between nine major components in CSAEs and the key regulatory proteins of four pathways known for their involvement in kidney injury. As shown in Figure 7 and Table S3, there were 14, 7, 13, and 12 proteins in the RhoA–ROCK pathway, apoptosis pathway, calcium regulation pathway, and necrosis pathway, respectively, which correspondingly generated 51, 9, 45, and 36 compound–protein complexes with a total score higher than 7 in these four pathways. Of note, the percentage of target proteins for the nine major components in the Rho and Rho-associated coiled-coil protein kinase (ROCK) pathway was greater than that in the other three pathways. The overall docking situation was shown in Table S4. These results indicate a direct interaction between the nine major components in CSAEs and the RhoA–ROCK pathway.

**Figure 8:**
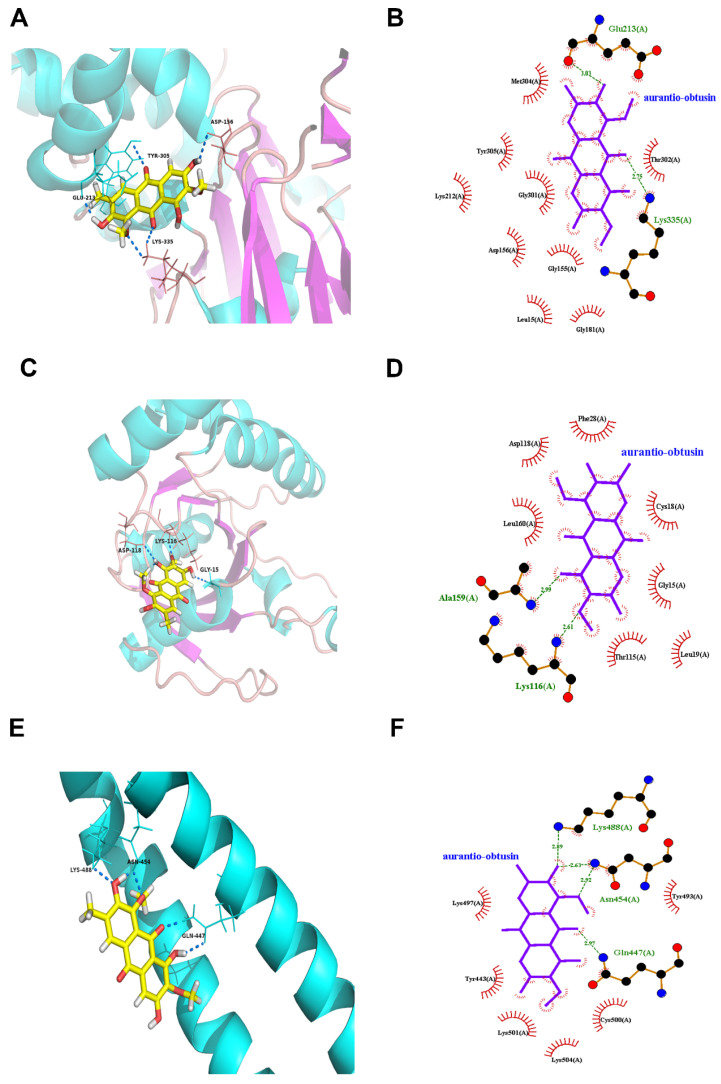
Molecular docking of aurantio-obtusin into the active sites of human F-actin (**A**,**B**), Rac1 (**C**,**D**), and occludin (**E**,**F**). The 3D structure is shown in panels (**A**,**C**,**E**), while the 2D structure is shown in panels (**B**,**D**,**F**).

**Figure 9:**
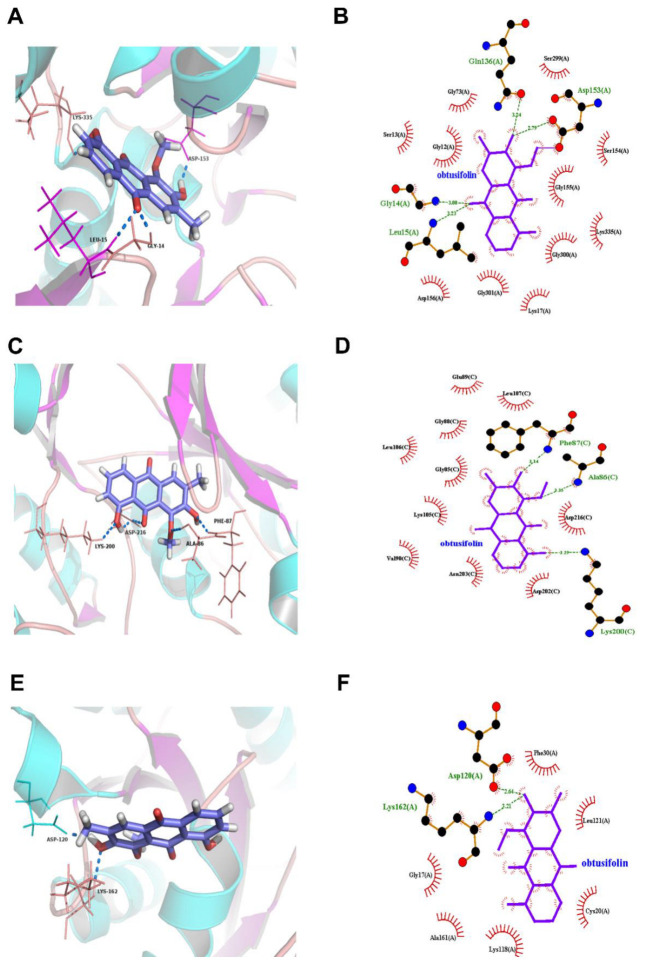
Molecular docking of obtusifolin into the active sites of human F-actin (**A**,**B**), Rock1 (**C**,**D**), and RhoA (**E**,**F**). The 3D structure is shown in panels (**A**,**C**,**E**), while the 2D structure is shown in panels (**B**,**D**,**F**).

**Figure 10:**
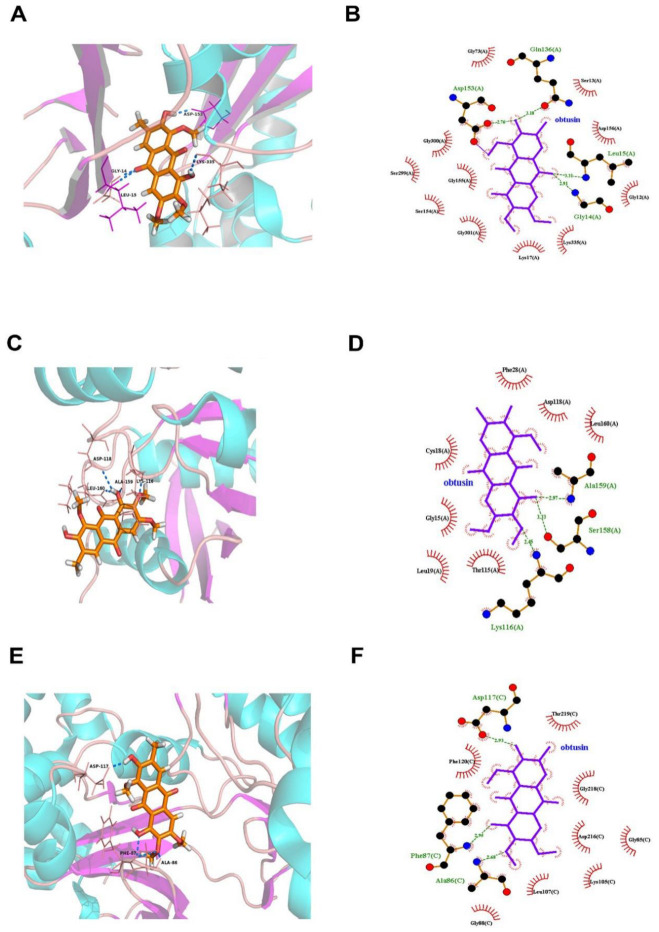
Molecular docking of obtusin into the active sites of human F-actin (**A**,**B**), Rac1 (**C**,**D**), and Rock1 (**E**,**F**). The 3D structure is shown in panels (**A**,**C**,**E**), while the 2D structure is presented in panels (**B**,**D**,**F**).

We next examined how the RhoA–ROCK pathway could possibly interact with the components of CSAEs. Since obtusifolin, aurantio-obtusin, and obtusin were the three most abundant components of CSAEs, we focused on these compounds for exemplary illustration. Intriguingly, all of obtusifolin, aurantio-obtusin, and obtusin were found to directly bind to F-actin, ROCK1, occludin, and Rac1 proteins through the formation of different degrees of hydrogen bonds and hydrophobic bonds (Figure 8, Figure 9 and Figure 10 and Table S5). Specifically, obtusin and aurantio-obtusin bonded with Rac1 via hydrogen bonds formed by the interaction with Ala159 (A) and Lys116 (A). Obtusin and obtusifolin bound to ROCK1 via hydrogen bonds formed by the interaction with Phe87 (C) and Ala86 (C). Obtusin and obtusifolin bound to F-actin via hydrogen bonds formed by interactions with Asp153 (A), Gln136 (A), Gly14 (A), and Leu15 (A), while aurantio-obtusin interacted with Lys335 (A) and Glu213 (A). Thus, these computational predictions imply that the RhoA–ROCK signaling pathway and F-actin could be potential targets of CSAEs.

### 3.4. F-Actin Protein Expression Was Inhibited in the Kidneys of CSAEs-Treated Rats

**Figure 11:**
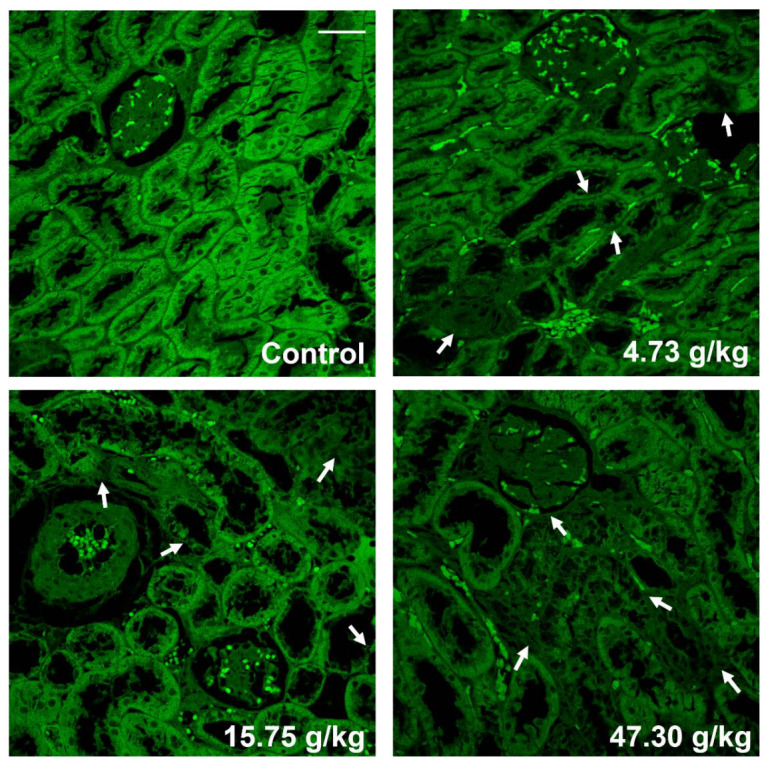
Immunofluorescence staining showing F-actin expression was inhibited in kidney tissues in CSAEs-treated rats. Alexa 488 phalloidin staining for F-actin (green) in the kidneys of male rats after administration of CSAEs for 28 days. The injury sites are indicated by white arrows. Scale bar = 50 μm; *n* = 8.

To validate the in silico prediction findings, F-actin expression was determined in rat kidneys after 28 days of administration of CSAEs. Results from immunofluorescence staining showed that compared to intact cytoskeleton and membrane structure as well as clear brush borders in the proximal tubules of control rats, F-actin expression was significantly suppressed in the kidneys of CSAEs-treated rats, which was accompanied by disrupted membrane and brush borders in renal cells (Figure 11). Together with computational prediction, these results identify that F-actin is a toxic action target of CSAEs.

### 3.5. The RhoA–ROCK Pathway Is Inhibited by CSAEs

**Figure 12:**
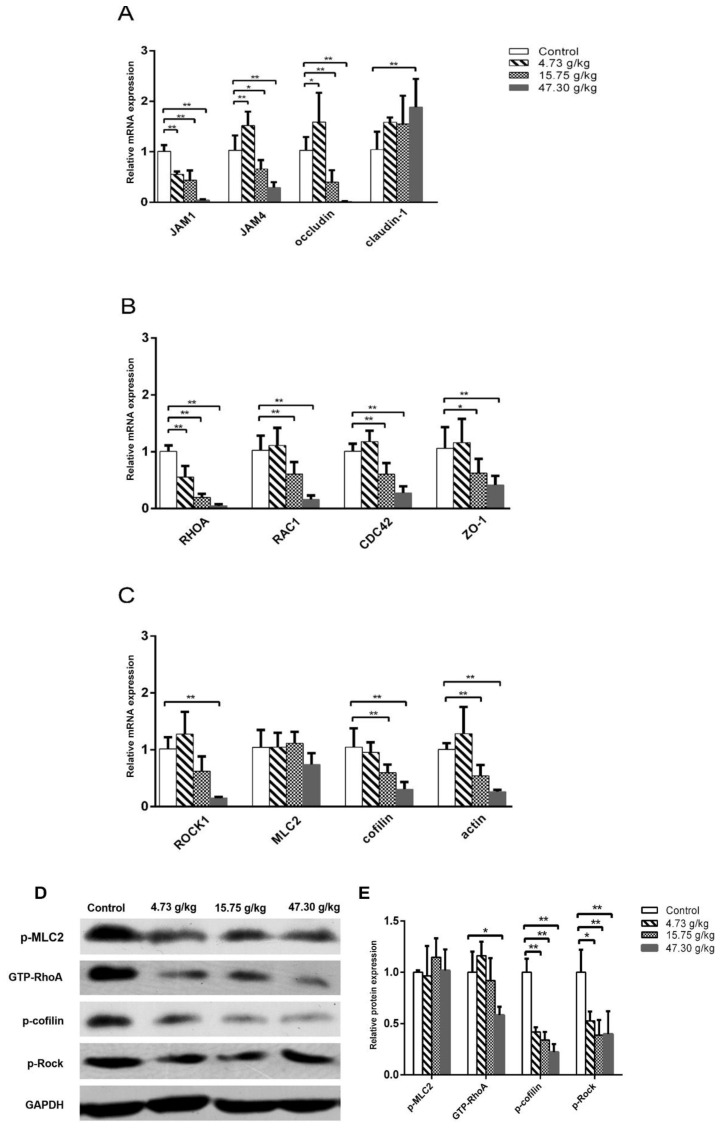
Effects of CSAEs on the expression of key regulatory genes of F-actin formation, assembly, and stabilization in the kidneys. Male SD rats were treated with CSAEs at the indicated doses for 28 days. The mRNA expression (**A**–**C**) was normalized to *Gapdh* mRNA, and protein expression (**D**) was normalized by GAPDH protein. Fold change (**E**) was calculated relative to untreated control rats. * and ** indicate *p* < 0.05 and *p* < 0.01, respectively, when compared with the vehicle control rats, *n* = 3.

To elucidate the molecular mechanisms mediating F-actin disruption by CSAEs, we focused on the RhoA–ROCK pathway, since cytoskeleton formation is partially controlled by the activity of this pathway and our in silico analyses suggested possible interactions between CSAEs and regulatory proteins of this pathway. Actin filament assembly/disassembly can be regulated by cofilins that integrate transmembrane signals to coordinate the spatial and temporal organization of actin filaments [24]. These transmembrane signals mainly involve cell–cell junctions, such as ZO-1, occludin, claudin, and JAMs [25]. As expected, the mRNA expressions of *RhoA*, *Rac1*, *ROCK1*, *actin*, *cofilin*, *CDC42*, *ZO-1*, and *JAM1* were significantly and dose-dependently downregulated in renal tissues of rats treated with higher doses of CSAEs (15.75 and 47.30 g/kg) (Figure 12A–C). Similar effects were also observed in *JAM4* and *occludin* mRNA expression, despite their mRNA levels being significantly induced in rats treated with low-dose CSAEs (4.73 g/kg), and the expression of *claudin-1* mRNA was also stimulated by CSAEs (Figure 12A).

In line with the mRNA expression profile, kidney tissue from CSAEs-treated rats expressed lower levels of GTP-RhoA protein and phosphorylated ROCK and cofilin proteins than that of control rats, while no changes were observed in phosphorylated levels of MLC2 protein (Figure 12D,E). The results suggest that CSAEs can inhibit the RhoA–ROCK–cofilin pathway, leading to disruption of actin depolymerization and F-actin stabilization and consequently renal toxicity.

## 4. Discussion

In earlier studies, Lee et al. [26] reported that *Cassia tora* Linn seed ethanol extract exhibited no treatment-related adverse effects, even at doses of over 2000 mg/kg/day in both sexes in rats, and no target organs were identified. Gao et al. [5] investigated the subchronic toxicity of ethanol extract of *Cassiae* semen. They detected increasing levels of BUN in 25, 35, and 45 g/kg B.W. dose groups and pigmentation in the kidney in 45 g/kg B.W. group, but they did not detect the main components of ethanol extract. Pei et al. [6] observed pigment deposition in the epithelial cells of the renal proximal convoluted tubules and atrophy or regeneration of renal tubules in SD rats after treatment with 10 g/kg B.W./day of freeze-dried powdered *Cassiae* semen, whose main compounds are anthraquinones, for 26 weeks. They also found that the male rats were more sensitive to freeze-dried powdered *Cassiae* semen than the female rats. Our findings were distinct from the observations of that study. In the present study, we obtained new findings in both renal effects and action mechanisms in rats treated with 4.73–47.30 g/kg B.W. of CSAEs for 28 days, and concluded that obtusifolin, aurantio-obtusin, and obtusin may disrupt the cytoskeleton by interacting with F-actin, Rac1, and ROCK1 after 28 days of administration of CSAEs.

The increase in Cr and BUN in serum indicated the risk of renal injury, while the increase in KIM-1, cystatin C, and β2M in urine and the changes in kidney histopathology indicated that CSAEs caused dysfunction of glomerular filtration and tubular damage. Brush borders, which are developed by kidney tubule epithelial cells, increase the cell surface and are required for the digestion and absorption of lumen components, which are vital to tubular reabsorption. Brush borders are mainly constituted by microvilli, and are single apical poles rich in cylindrical membrane protrusions. Each microvillus has an actin bundle backbone that is composed of 19 actin filaments [27]. F-actin staining demonstrated the disappearance of the brush border, which indicated that dysregulation of the actin cytoskeleton could be the main cause of kidney injury after 28 days of CSAEs administration.

Few experimental studies have shed new light on molecular and cellular mechanisms of *Cassiae* semen-induced nephrotoxicity. Huang et al. found that the mitochondrial pathway is involved in the nephrotoxicity induced by rhein, emodin, and aurantio-obtusin [28]. However, our previous study indicated that obtusifolin, aurantio-obtusin, and obtusin have high plasma concentrations and can accumulate in rat plasma after 28 days of administration of CSAEs [7]. In addition, apoptosis and necrosis are often related to mitochondrial dysfunction in the kidney [29], suggesting that the apoptosis pathway and necrosis pathway may not be core pathways. Therefore, obtusifolin, aurantio-obtusin, and obtusin were regarded as major nephrotoxicity components in CSAEs and may be involved in another pathway. In this study, according to the results of the composition–target interaction network model, most key regulatory proteins are related to the Rho–ROCK pathway, and actin is closely related to the RhoA–ROCK pathway.

We first explored the effects of *Cassiae* semen on regulating the actin cytoskeleton using molecular docking. The results indicated that the components in *Cassiae* semen are more likely to directly regulate the actin cytoskeleton through interaction between the key proteins in the RhoA–ROCK pathways, especially the proteins F-actin, Rac1, ROCK1, and occludin. The RhoA–ROCK signaling has been extensively investigated since it is critically involved in cell growth, differentiation, migration, cell contraction, adhesion, inflammation, and survival from apoptosis [30,31]. Rho GTPases act as molecular switches in the cell by cycling between the inactive GDP-bound state and the active GTP-bound state. The activation of RhoA stimulates ROCK, the downstream effector that phosphorylates LIM kinase (LIMK), myosin light chain (MLC), and cofilin, subsequently impacting some cellular processes [32], such as actin cytoskeleton remodeling, cell adhesion and migration, reactive oxygen species (ROS) formation, and cell apoptosis [33]. A study showed that inactivation of the Rho–ROCK1 pathway was associated with significant improvement in proteinuria and tubulointerstitial fibrosis, and could affect kidney function in small congenic regions (e.g., RBF and GFR) [34]. Another study revealed that in canine kidney cells, apoptosis could be induced through mitochondrial malfunction and cytoskeleton disassembly [21].

Therefore, we turned to the molecular level to see whether the RhoA–ROCK pathway played the most important role in our model. Results of RT-PCR and Western blot showed that GTP-RhoA, and p-cofilin, but not p-MLC-2, were downregulated at the protein level, while mRNAs related to upstream proteins (ZO-1, RAC1, JAM1, JAM4, occludin, claudin, and CDC42) were downregulated as well. This indicated that *Cassiae* semen preferred to impact the activity of cofilin rather than MLC2. It has reported that ROCK can promote the increase of intracellular Ca^2+^ concentration through the activation of Ca^2+^/calmodulin-dependent MLCK, resulting in the phosphorylation of MLC2 [35]. Thus, we speculate that the calcium regulation pathway can also be a secondary pathway. Whether cofilin promotes actin assembly or disassembly depends on the concentration of cofilin relative to actin and the relative concentrations of other actin-binding proteins. When the ratio of cofilin/actin subunits in the filament is low (less than 1%), persistent filament severing occurs. Cofilin severs rapidly but transiently at higher cofilin/actin molar ratios because it binds to F-actin cooperatively and stabilizes F-actin in a twisted form as it saturates the severed pieces [36]. As an actin depolymerizing factor, cofilin can be blocked by activating the RhoA–LIMK–cofilin pathway [37], and dephosphorylation of cofilin enhances the activation to induce apoptosis [38]. In the present study, we demonstrated that *Cassiae* semen can interact with transmembrane signals, impact the RhoA–ROCK pathway, and lead to a change in cofilin and actin cytoskeleton, and that the main components of CSAEs can influence actin by the RhoA–ROCK pathway or bind directly to F-actin. It has been shown that the Rho family small G protein Rac–GTP signaling pathway phosphorylates LIMK1 at Thr508 and LIMK2 at Thr505 by p21-activated kinases 1 and 4, which further led to phosphorylation of cofilin at Ser3 [39]. Activated cofilin converts the polymer F-actin into monomer G-actin, which in turn affects the formation of the cytoskeleton. The loss of p-cofilin decreased the stability of F-actin. However, the exact molecular initiation event is still worthy of further investigation because CSAEs are a mixture. Our findings indicate the disruption of actin as a key event of CSAEs-mediated nephrotoxicity in rats.

**Figure 13:**
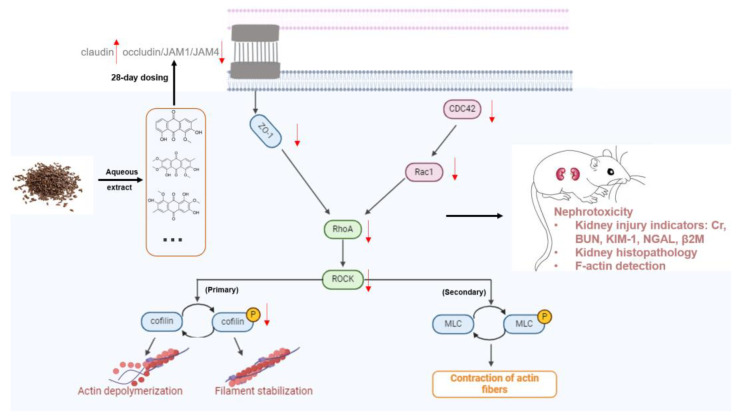
Schematic illustration showing dysregulation of actin dynamics by CSAEs via the RhoA–ROCK signaling pathway.

In conclusion, we demonstrated that *Cassiae* semen can cause nephrotoxicity after 28 days of repeated administration in rats and that the potential action mechanisms were the abnormal structure of glomeruli and tubules, especially tubules, which manifested as the disappearance of F-actin (Figure 13). Taking together the results of molecular docking and the nephrotoxicity mechanism, *Cassiae* semen impacts actin cytoskeleton depolymerization and filament stabilization by interacting with the RhoA–ROCK pathway and/or directly binding to F-actin.

## Data Availability

All data are available upon reasonable request to the corresponding author.

## Supplementary Materials

The following supporting information can be downloaded at https://www.mdpi.com/article/10.3390/toxics12080556/s1. Table S1: Primer sequences; Table S2: The meaning of kidney injury indicators; Table S3: The target proteins in different renal injury pathways; Table S4: The interactions between 9 major components and 4 kidney injury pathways by molecular docking; Table S5: Molecular interactions between 3 anthraquinones extracted from Cassiae Semen and proteins of RhoA–ROCK.
