# Supplementary material for: Actin Dysregulation Mediates Nephrotoxicity of Cassiae Semen Aqueous Extracts
Source: Toxics. 2024 Jul 30;12(8):556. doi: 10.3390/toxics12080556 (PMC11360101; doi:10.3390/toxics12080556)
Supplement: Supplementary file 1 [file toxics-12-00556-s001.zip › toxics-3054858-supplementary.docx]

**Table S1.** Primer sequences.

| Gene name | Primer sequences | |
| --- | --- | --- |
|  | Forward | Reverse |
| Ctnnb1 | 5’- GTCTGAGGACAAGCCACAGGACTAC-3’ | 5’- AATGTCCAGTCCGAGATCAGCA-3’ |
| Gapdh | 5’- GGCACAGTCAAGGCTGAGAATG-3’ | 5’- ATGGTGGTGAAGACGCCAGTA-3’ |
| Rock1 | 5’- TGCCAATAGTCCTTGGGTTGTTC-3’ | 5’- CAAGGTCTCCACCAGGCATGTA-3’ |
| Ocln | 5’- GTCTTGGGAGCCTTGACATCTTG-3’ | 5’- GCATTGGTCGAACGTGCATC-3’ |
| Mylpf | 5’- GAGCTACTGCCTTGCCCTCA -3’ | 5’- TGTCAATAATGCCATCCCTGTTC-3’ |
| F11r | 5’- CGCGTCGGGATTGAAACTG-3’ | 5’- GAACCCTTGCCTTGCACCA-3’ |
| Rhoa | 5’- CAGCAAGGACCAGTTCCCAGA-3’ | 5’-AGCTGTGTCCCATAAAGCCAACTC-3’ |
| Rac1 | 5’- CCTGCTCATCAGTTACACGACCA-3’ | 5’- GTCCCAGAGGCCCAGATTCA-3’ |
| Cdc42 | 5’- CGGGATCTGAAGGCTGTCAAGTA-3’ | 5’- AGGCTCCAAGGCAGCCAATA-3’ |
| Igsf5 | 5’- AAGCAGATCCCGAGACATGGTTA-3’ | 5’- GGCTACTGCGTTGTTCTGGAAG-3’ |
| Tjp1 | 5’- CCATCTTTGGACCGATTGCTG-3’ | 5’- TAATGCCCGAGCTCCGATG-3’ |
| Cldn2 | 5’- TTTCCGCTGGAGCATCTCT-3’ | 5’- TGTCTCTGGCAAGCTGACTTCT-3’ |
| Actg1 | 5’- CCGGCTTACACTGCGTTTC-3’ | 5’- ACATGCCGGAGCCATTGTC-3’ |
| Cfl1 | 5’- CAAGATGCTGCCAGACAAGGAC-3’ | 5’- GCATCCTTGGAGCTGGCATAG-3’ |

**Table S2.** The meaning of kidney injury indicators.

| Biomarkers | Injury sites | featues | Reference |
| --- | --- | --- | --- |
| Calbindin | Distal tubules, collecting ducts | It is a cytosolic calcium-binding protein which plays a role in calcium reabsorption and localizes primarily to distal tubules and collecting ducts. Calbindin concentrations are elevated in urine following kidney injury. | Sooy, K. et al., 2000; M Takashi et al., 1996 |
| KIM-1 | Proximal convoluted tubule | It is a transmembrane glycoprotein of renal proximal tubule epithelial cells which are slightly expressed in normal liver, kidney and spleen, but upregulated in acute kidney injury, renal fibrosis and other preclinical and clinical models. | de Geus et al., 2012 |
| Osteopontin (OPN) | All tubular segments and glomeruli | Osteopontin (OPN) is an extracellular matrix protein produced primarily by the distal nephron and secreted into urine in the normal kidney. It attracts macrophages and participates in the development of renal fibrosis after kidney injury and could be used as mid-term injury indicator (14 days). | Carla, Patrícia, Carlos, et al., 2014; Xie Y et al., 2001 |
| β-2-Microglobulin (β2M) | Proximal convoluted tubule; glomerulus | The monomer form ofβ2M can be filtered through glomerulus and reabsorbed by proximal convoluted tubular cells; limited in clinical application due to its instability in acidic urine. | Schaub et al., 2005 |
| Cystatin C | Proximal convoluted tubule; glomerulus; distal convoluted tubules; collecting duct | Cystatin C is mainly distributed in extracellular fluid, with the lowest concentration in urine in normal. The increase of Cystatin C in urine indicates the dysfunction of renal tubular. | Pianta et al., 2017 |
| NGAL/Lipocalin-2 | Renal tubule; collecting duct | In ischemic or nephrotoxic kidney injury, NGAL is expressed in large quantities by the kidneys and is released into urine and plasma. The content of NGAL increases within 2 hours after injury, making it an early and sensitive biomarker of kidney injury | Avci Çiçek et al., 2016 |

**Table S3.** The proportion of target proteins in different renal injury pathways.

|  | RhoA-ROCK | | Apoptosis | | Necrosis | | Calcium Regulation | |
| --- | --- | --- | --- | --- | --- | --- | --- | --- |
|  | Number of target protein | % target protein | Number of target protein | % target protein | Number of target protein | % target protein | Number of target protein | % target protein |
| rhein | 3 | 25．00 | 0 | 0 | 1 | 8．33 | 3 | 23．08 |
| emodin | 3 | 25.00 | 1 | 14．29 | 2 | 16．67 | 2 | 15．38 |
| OMD | 6 | 50.00 | 0 | 0 | 2 | 16．67 | 5 | 38．46 |
| cassiaside | 10 | 83.33 | 3 | 42．86 | 8 | 66．67 | 8 | 61．54 |
| questin | 3 | 25．00 | 1 | 14．29 | 2 | 16．67 | 2 | 15．38 |
| obtusin | 7 | 58.33 | 2 | 28．57 | 6 | 50．00 | 4 | 30．77 |
| aurantio-obtusin | 6 | 50.00 | 1 | 14．29 | 3 | 25．00 | 5 | 38．46 |
| obtusifolin | 3 | 25．00 | 0 | 0 | 1 | 8．33 | 2 | 15．38 |
| EDG | 10 | 83．33 | 1 | 14．29 | 11 | 91．67 | 6 | 46．15 |


**Table S4.** The interactions between 9 major components and 4 kidney injury pathways by molecular docking.

| Kidney injury pathways | Protein | PDB ID | 9 major components in AECS | | | | | | | | |
| --- | --- | --- | --- | --- | --- | --- | --- | --- | --- | --- | --- |
|  |  |  | NEW | Rhein | Emodin | OMD | Cassiaside | Questin | Obtusin | Aurantio-obtusin | Obtusifolin |
| RhoA-ROCK | Jam1 | 1NBQ | 8.47 | 5.88 | 5.38 | 5.34 | 4.74 | 4.74 | 4.57 | 4.36 | 4.25 |
|  | Occludin | 1WPA | 9.00 | 4.59 | 6.38 | 6.88 | 7.64 | 5.20 | 8.65 | 8.15 | 4.97 |
|  | Cdc42 | 2KB0 | 9.03 | 4.74 | 5.45 | 7.57 | 7.77 | 5.45 | 8.38 | 6.16 | 5.01 |
|  | Rac1 | 2RMK | 7.81 | 7.04 | 6.11 | 7.69 | 7.69 | 7.93 | 10.48 | 8.85 | 5.67 |
|  | ZO-1 | 4OEO | 5.22 | 3.86 | 5.91 | 5.47 | 7.23 | 4.53 | 4.85 | 4.75 | 4.20 |
|  | Claudin-1 | 4YYX-A | 8.26 | 5.70 | 6.29 | 6.19 | 7.89 | 6.10 | 5.77 | 6.95 | 6.69 |
|  | Claudin-2 | 4YYX-B | 7.94 | 7.36 | 6.60 | 7.23 | 7.72 | 6.10 | 7.94 | 8.05 | 6.78 |
|  | Rock1 | 5WNG | 9.57 | 6.27 | 7.36 | 7.53 | 9.47 | 6.13 | 9.07 | 7.33 | 8.01 |
|  | RhoA | 5FR1 | 9.06 | 6.54 | 6.65 | 8.28 | 10.51 | 7.61 | 7.57 | 8.02 | 7.34 |
|  | MLC2 | 5TBY | 7.08 | 5.44 | 5.23 | 6.67 | 8.67 | 6.48 | 5.65 | 5.40 | 6.28 |
|  | Catenin beta-1 | 4DJS | 7.29 | 6.32 | 7.52 | 6.58 | 10.57 | 6.44 | 6.72 | 6.90 | 6.34 |
|  | cofilin | 5L6W | 5.55 | 3.80 | 2.83 | 2.77 | 4.33 | 3.26 | 4.93 | 6.20 | 2.89 |
|  | F-actin | 3W3D | 11.28 | 9.43 | 9.50 | 8.34 | 10.90 | 7.71 | 9.91 | 9.53 | 9.78 |
|  | cadherin | 4ZML | 5.82 | 2.85 | 4.08 | 4.45 | 4.16 | 3.07 | 4.62 | 2.72 | 3.27 |
| Apoptosis | AKT | 4GV1 | 8.51 | 5.62 | 6.80 | 6.97 | 10.63 | 7.84 | 8.24 | 7.92 | 6.78 |
|  | PI3K | 4WAF | 6.95 | 6.91 | 7.04 | 6.76 | 8.69 | 6.84 | 8.37 | 6.70 | 6.74 |
|  | Cytochrome C | 1J3S | 4.51 | 4.73 | 4.94 | 6.44 | 9.86 | 6.05 | 3.92 | 3.91 | 2.86 |
|  | Bcl-2 | 5VAY | 6.99 | 5.39 | 5.05 | 4.94 | 5.65 | 5.87 | 6.55 | 5.21 | 5.59 |
|  | Caspase-9 | 4RHW | 6.82 | 5.70 | 4.72 | 5.09 | 6.44 | 3.89 | 3.97 | 4.76 | 6.38 |
|  | Caspase-3 | 2DKO | 4.52 | 4.76 | 4.52 | 4.28 | 5.85 | 4.45 | 5.29 | 4.67 | 5.04 |
|  | Mtor | 4DRH | 5.37 | 4.72 | 6.17 | 5.55 | 6.93 | 4.14 | 5.56 | 5.70 | 5.79 |
| Calcium Regulation | PLCγ | 4EY0 | 9.82 | 7.84 | 6.07 | 6.83 | 8.55 | 6.22 | 7.22 | 7.38 | 6.42 |
|  | ORAI1 | 2MAK | 5.05 | 3.93 | 4.65 | 5.00 | 5.46 | 4.99 | 4.73 | 4.42 | 5.34 |
|  | RYR2 | 4JKQ | 9.89 | 5.75 | 6.16 | 7.20 | 8.53 | 6.85 | 7.77 | 8.26 | 6.07 |
|  | DAO | 4QFC | 11.91 | 7.67 | 9.21 | 8.32 | 9.39 | 8.62 | 8.98 | 8.27 | 8.83 |
|  | PKA | 6C0U | 8.60 | 7.26 | 7.71 | 8.74 | 9.01 | 7.27 | 7.07 | 6.25 | 9.50 |
|  | TRPC3 | 6CUD | 7.93 | 5.15 | 5.79 | 7.73 | 6.38 | 4.55 | 5.51 | 5.37 | 5.22 |
|  | STIM1 | 2MAK | 5.11 | 6.98 | 4.90 | 6.10 | 7.01 | 4.58 | 6.02 | 5.59 | 5.82 |
|  | PLN | 2HYN | 5.86 | 6.79 | 5.88 | 5.85 | 6.39 | 5.78 | 5.25 | 7.03 | 4.86 |
|  | STIM2 | 2L5Y | 5.10 | 3.58 | 3.82 | 3.43 | 7.25 | 5.27 | 4.18 | 3.81 | 4.75 |
|  | MLCK1 | 2YR3 | 3.88 | 2.89 | 3.38 | 2.75 | 3.67 | 3.42 | 3.02 | 3.42 | 4.07 |
|  | NCX/TLX2 | 3A03 | 7.12 | 6.19 | 5.55 | 7.15 | 8.23 | 5.70 | 6.10 | 6.55 | 6.81 |
|  | SERCA1 | 3BA6 | 11.67 | 10.41 | 8.01 | 7.90 | 13.01 | 8.95 | 10.14 | 9.24 | 7.86 |
|  | PLCβ | 3OHM | 6.07 | 6.04 | 6.33 | 5.91 | 8.28 | 4.52 | 6.38 | 5.39 | 6.20 |
| necrosis | TNFR1 | 1NCF | 7.11 | 6.57 | 6.14 | 6.31 | 8.25 | 5.06 | 7.36 | 7.51 | 5.59 |
|  | TRAM | 2M1W | 8.78 | 5.32 | 6.54 | 6.65 | 7.91 | 6.00 | 6.69 | 6.52 | 5.80 |
|  | TRIF | 4COM | 12.51 | 6.89 | 7.29 | 5.62 | 7.08 | 6.67 | 7.84 | 6.50 | 5.60 |
|  | TLR4 | 4G8A | 5.75 | 7.06 | 6.31 | 6.35 | 7.30 | 4.98 | 5.85 | 6.85 | 5.27 |
|  | Casp8 | 4JJ7 | 8.56 | 5.95 | 4.87 | 6.39 | 6.72 | 6.52 | 7.54 | 5.98 | 6.70 |
|  | MLKL | 4M67 | 12.09 | 5.93 | 7.61 | 7.23 | 9.17 | 7.42 | 10.35 | 8.02 | 7.76 |
|  | RIPK3 | 5V7Z | 7.74 | 3.81 | 3.75 | 4.53 | 5.64 | 4.41 | 5.45 | 5.52 | 5.74 |
|  | TRADD | 5XME | 7.10 | 4.30 | 3.91 | 4.57 | 5.18 | 3.10 | 5.59 | 4.92 | 4.73 |
|  | RIPK1 | 6C3E | 9.51 | 5.74 | 6.77 | 7.51 | 9.92 | 7.38 | 7.50 | 7.39 | 6.06 |
|  | TLR3 | 5GS0 | 7.40 | 3.86 | 4.46 | 5.49 | 8.29 | 3.64 | 7.25 | 5.49 | 5.05 |
|  | TRAF2 | 1CZY | 8.74 | 4.60 | 4.58 | 6.34 | 7.59 | 4.41 | 6.42 | 5.27 | 5.27 |
|  | FADD | 1E3Y | 8.46 | 4.00 | 4.58 | 5.73 | 6.51 | 4.83 | 6.28 | 6.34 | 5.26 |

| Component | Target Protein | PDB ID | Total score | Crash | Polar | Strain | Hydrophobic bond number | Hydrogen bond number | Residues involved in hydrogen bond formation |
| --- | --- | --- | --- | --- | --- | --- | --- | --- | --- |
| Obtusin | Rac1 | 2RMK | 10.48 | -2.02 | 5.14 | 2.14 | 7 | 3 | Ala159(A), Lys116(A), Ser158(A) |
|  | F-actin | 3W3D | 9.91 | -1.11 | 3.98 | 1.25 | 11 | 4 | Asp153(A),Gln136(A),Gly14(A),Leu15(A) |
|  | Rock1 | 5WNG | 9.07 | -1.01 | 3.08 | 0.86 | 8 | 3 | Phe87(C), Asp117(C), Ala86(C) |
| Obtusifolin | F-actin | 3W3D | 9.78 | -1.07 | 4.15 | 0.75 | 11 | 4 | Asp153(A), Gln136(A), Gly14(A), Leu15(A) |
|  | Rock1 | 5WNG | 8.01 | -1.00 | 4.64 | 1.17 | 10 | 3 | Phe87(C), Lys200(C), Ala86(C) |
|  | RhoA | 5FR1 | 7.34 | -1.56 | 3.63 | 0.66 | 6 | 2 | Asp120(A), Lys162(A) |
| Aurantio-obtusin | F-actin | 3W3D | 9.53 | -1.52 | 4.87 | 0.73 | 9 | 2 | Lys335(A), Glu213(A) |
|  | Rac1 | 2RMK | 8.85 | -1.86 | 5.62 | 1.06 | 7 | 2 | Ala159(A), Lys116(A) |
|  | Occludin | 1WPA | 8.15 | -1.20 | 4.68 | 1.53 | 6 | 4 | Lys488(A), Asn454(A), Gln447(A) |

**Table S5.** Molecular interactions between 3 anthraquinones extracted from *Cassiae* Semen and proteins of RhoA-ROCK.
